# Sex Differences in Heart Failure Following Acute Coronary Syndromes

**DOI:** 10.1016/j.jacadv.2023.100294

**Published:** 2023-04-26

**Authors:** Edina Cenko, Olivia Manfrini, Jinsung Yoon, Mihaela van der Schaar, Maria Bergami, Zorana Vasiljevic, Guiomar Mendieta, Goran Stankovic, Marija Vavlukis, Sasko Kedev, Davor Miličić, Lina Badimon, Raffaele Bugiardini

**Affiliations:** a Laboratory of Epidemiological and Clinical Cardiology, Department of Medical and Surgical Sciences, University of Bologna, Bologna, Italy; b IRCCS Azienda Ospedaliero-Universitaria di Bologna Sant’Orsola Hospital, Bologna, Italy; c Google Cloud AI, Sunnyvale, California, USA; d Department of Electrical and Computer Engineering, University of California, Los Angeles, Los Angeles, California, USA; e Department of Applied Mathematics and Theoretical Physics and Department of Population Health, Cambridge Centre for Artificial Intelligence in Medicine, University of Cambridge, Cambridge, United Kingdom; f Medical Faculty, University of Belgrade, Belgrade, Serbia; g Centro Nacional de Investigaciones Cardiovasculares Carlos III (CNIC), Madrid, Spain; h Clinic of Cardiology, University Clinical Centre of Serbia, Belgrade, Serbia; i University Clinic for Cardiology, Skopje, Republic of North Macedonia; j Faculty of Medicine, Ss. Cyril and Methodius University in Skopje, Skopje, Republic of North Macedonia; k Department for Cardiovascular Diseases, University Hospital Center Zagreb, University of Zagreb, Zagreb, Croatia; l Cardiovascular Research Program ICCC, IR-IIB Sant Pau, Hospital de la Santa Creu i Sant Pau, CiberCV-Institute Carlos III, Barcelona, Spain

**Keywords:** acute heart failure, outcomes, sex differences

## Abstract

**Background:**

There have been conflicting reports regarding outcomes in women presenting with an acute coronary syndrome (ACS).

**Objectives:**

The objective of the study was to examine sex-specific differences in 30-day mortality in patients with ACS and acute heart failure (HF) at the time of presentation.

**Methods:**

This was a retrospective study of patients included in the International Survey of Acute Coronary Syndromes-ARCHIVES (ISACS-ARCHIVES; NCT04008173). Acute HF was defined as Killip classes ≥2. Participants were stratified according to ACS presentation: ST-segment elevation myocardial infarction (STEMI) and non-ST-segment elevation ACS (NSTE-ACS). Differences in 30-day mortality and acute HF presentation at admission between sexes were examined using inverse propensity weighting based on the propensity score. Estimates were compared by test of interaction on the log scale.

**Results:**

A total of 87,812 patients were included, of whom 30,922 (35.2%) were women. Mortality was higher in women compared with men in those presenting with STEMI (risk ratio [RR]: 1.65; 95% CI: 1.56-1.73) and NSTE-ACS (RR: 1.18; 95% CI: 1.09-1.28; *P*_interaction_ <0.001). Acute HF was more common in women when compared to men with STEMI (RR: 1.24; 95% CI: 1.20-1.29) but not in those with NSTE-ACS (RR: 1.02; 95% CI: 0.97-1.08) (*P*_interaction_ <0.001). The presence of acute HF increased the risk of mortality for both sexes (odds ratio: 6.60; 95% CI: 6.25-6.98).

**Conclusions:**

In patients presenting with ACS, mortality is higher in women. The presence of acute HF at hospital presentation increases the risk of mortality in both sexes. Women with STEMI are more likely to present with acute HF and this may, in part, explain sex differences in mortality. These findings may be helpful to improve sex-specific personalized risk stratification.

The association between sex and outcomes after myocardial infarction has been extensively studied^1^; however, the reasons for these disparities are still not completely understood.^2^ Previous studies yielded mixed results.^1^^,^^3^, ^4^, ^5^, ^6^, ^7^ Some reports have suggested that older age and lower rate of coronary interventions in women might explain the disparity in outcomes. Others have shown that differences may be due to the pathophysiology of coronary heart disease in women.^7^ Some studies have shown that the higher mortality in women is restricted to patients with persistent ST-segment elevation myocardial infarction (STEMI), with no sex differences in patients with non-ST-segment elevation acute coronary syndromes (NSTE-ACS).^4^^,^^6^, ^7^, ^8^ Finally, acute heart failure (HF), a common complication of ACS, can result in a several-fold increase in mortality and this may explain sex differences in mortality^8^, ^9^, ^10^, ^11^; prior studies did not control for acute HF on hospital admission, and specifically lacked information on HF phenotyping. Left ventricular (LV) function is a strong predictor of mortality and is known to differ between men and women. Therefore, the objective of this study was to analyze a large European cohort to assess sex differences in the acute HF presentation complicating ACS and 30-day mortality using propensity score-based analytic methods in groups of patients with comparable severity of disease and therapeutic targets, specifically with STEMI and NSTE-ACS.

## Methods

### Setting and design

The ISACS (International Survey of Acute Coronary Syndromes) Archives (NCT04008173) is part of ISACS (NCT01218776) registry. Details of the study design, sampling, and recruitment have been previously published.^7^^,^^12^ Recruiting date of the current study is from January 2003 to January 2019. In brief, the registry included data from 41 centers in 11 European countries: Bosnia and Herzegovina, Croatia, Italy, Kosovo, Lithuania, Macedonia, Hungary, Moldova, Montenegro, Romania, and Serbia. Among these sites, there were 22 tertiary health care services providing percutaneous coronary intervention (PCI).^12^ This study complies with the Declaration of Helsinki. The local research ethics committee from each hospital approved the study. Because patient information was collected anonymously, institutional review boards waived the need for individual-informed consent. All data were transferred to the Department of Electrical and Computer Engineering, University of California, Los Angeles, where final statistical analyses were done.

### Patient population

Patients were eligible for this study if they had clinically confirmed ACS. We excluded patients with incomplete data, resulting in a final study population of 87,812 patients (Supplemental Figure 1). Comparison of the baseline characteristics of the excluded and included populations are shown in the Supplemental Methods. Participants were stratified by ACS subtypes: STEMI and NSTE-ACS. There were 56,038 STEMI and 31,774 NSTE-ACS. Patients were identified from hospital records. Diagnosis was validated by 2 cardiologists based on the presence of symptoms plus electrocardiogram changes and biomarker release indicative of myocardial infarction.^7^^,^^13^

### Outcome

The primary outcome was 30-day all-cause mortality from hospital admission. The 30-day window was selected to enrich the data over that acquired during the index hospitalization while mitigating survivor bias. The secondary outcome was the risk of acute HF on admission.

### Study definitions and data collection

The diagnosis of acute HF was based on clinical symptoms or signs and radiographic evidence of pulmonary congestion. Acute HF was defined as Killip class ≥2. Data were also collected on the use of reperfusion therapies (PCI and/or fibrinolysis) and the type of medications given on hospital admission: aspirin, P2Y_12_ inhibitors, heparins, (unfractionated heparin, low molecular weight heparin, fondaparinux), glycoprotein IIb/IIIa inhibitors, nitrates (nitroglycerin, nitroprusside), diuretics (furosemide, torsemide, bumetanide), inotropic agents (dopamine, dobutamine, milrinone) and digoxin; or during hospitalization: angiotensin-receptor blockers (candesartan, valsartan, losartan), angiotensin-converting enzyme inhibitors (captopril, enalapril, lisinopril, ramipril, trandolapril), and beta-blockers. Time to hospital presentation was calculated from the date and time of symptom onset to the date and time of hospital arrival. Time to hospital presentation was presented as a dichotomous variable: delayed (≥120 minutes) vs early (<120 minutes) presentation according to the American College of Cardiology/American Heart Association practice guidelines.^14^ Emergency coronary artery bypass graft was performed as need for surgery after PCI, and as so, outcomes were included in the subgroup of patients with PCI.

### Echocardiographic study

We assessed LV ejection fraction (EF) using transthoracic echocardiography at discharge in those patients who survived the index event. Acute HF with reduced EF (HFrEF) was diagnosed when EF was ≤40% according to guidelines.^15^

### Statistical analysis

Baseline characteristics were reported as percentages for categorical variables and means with standard deviation for continuous variables (Table 1). Baseline characteristics, treatment, and clinical outcomes between women and men were compared. We used Multiple Imputation using Chained Equation algorithm as imputation method to treat missing data (Supplemental Methods). Estimates of the odds ratios (ORs) and associated 95% CIs were obtained with the use of multivariable logistic regressions. Baseline covariates included demographics, cardiovascular risk factors, medical history, and clinical features on hospital presentation (Table 1). Estimates were also performed using a parametric balancing strategy by inverse probability weighting. Inverse probability weights were calculated using the propensity score to create a sample in which the distribution of measured baseline covariates was independent from sex (Supplemental Methods). Because of the instability that can be induced by extreme weights, stabilized weights were used that also preserve the original sample size. We created a threshold for weights to avoid the impacts of the outliers. We used 0.01 as the threshold of the propensity weighting. Standardized differences after weighting were calculated to ensure balanced treatment groups with respect to baseline characteristics. Groups were considered balanced when the standardized difference was <10% (Supplemental Methods). Baseline covariates in the inverse probability weighting models included demographics, cardiovascular risk factors, medical history, and clinical features on hospital presentation (Table 1). We calculated women-to-men risk ratios (RRs) with their 95% CIs (Supplemental Methods). Analyses through inverse probability weighting were applied to each observation level. All models were stratified by type of myocardial infarction: STEMI and NSTE-ACS. The main outcome measures were rates of acute HF on admission and 30-day mortality.

**Table 1:** Clinical Factors and Outcomes Stratified by Sex in Patients With Acute Coronary Syndrome: Inverse Probability Weighting

	Women (n = 30,922)	Men (n = 56,890)	Standardized Difference
Age (y)	63.4 ± 12.1	63.6 ± 11.7	−0.02
Cardiovascular risk factors			
Family history of CAD	43.8	43.6	0.004
Diabetes	25.7	25.9	−0.005
Hypertension	69.4	69.6	−0.005
Hypercholesterolemia	50.3	50.7	−0.008
Current smokers	42.7	42.3	0.009
Former smokers	1.9	1.8	0.004
Clinical history of CHD			
Prior stable angina	28.7	28.6	0.002
Prior MI	19.4	19.0	0.009
Prior PCI	8.7	8.5	0.007
Prior CABG	4.0	3.7	0.02
Clinical history of CVD			
Peripheral artery disease	5.4	5.3	0.005
Prior stroke	5.0	4.9	0.003
Clinical presentation at admission			
ST-segment shifts in anterior leads (at electrocardiogram)	43.4	43.2	0.004
SPB at admission, mm Hg	137.1 ± 28.8	137.2 ± 27.7	−0.006
Heart rate at admission, beats/min	82.4 ± 21.7	82.4 ± 21.2	−0.003
Serum creatinine levels at admission, mg/dL	1.3 ± 1.4	1.2 ± 0.9	0.09

Outcomes			*P* Value
Primary outcome: 30-d mortality	12.9	9.4	<0.001
Risk ratio (95% CI)	1.43 (1.37-1.50)		<0.001
Secondary outcome: acute heart failure	30.2	27.6	<0.001
Risk ratio (95% CI)	1.13 (1.10-1.17)		<0.001

Values are mean ± SD or % unless otherwise indicated. SI conversion factor: To convert serum creatinine to mmol/L, multiply by 88.4.

ACS = acute coronary syndrome; CABG = coronary artery bypass graft; CAD = coronary artery disease; CHD = coronary heart disease; CVD = cardiovascular disease; MI = myocardial infarction; PAD = peripheral artery disease; PCI = percutaneous coronary intervention; SBP = systolic blood pressure.

Sensitivity analyses were conducted to evaluate the importance of medications administered on admission or during hospital stay on sex differences in outcomes. Two models were run that incrementally added covariates. The first model included clinical characteristics as reported in Table 1 and medications administered on admission (nitrates, inotropes, diuretics, digoxin, aspirin, clopidogrel, heparins, glycoprotein IIb/IIIa inhibitors). Model 2 included additional medications administered during hospital stay (angiotensin-converting enzyme inhibitors/angiotensin-receptor blockers, beta-blockers). A separate analysis was done for patients undergoing reperfusion therapies (primary PCI and fibrinolysis). Analyses on the rates of acute HF were also performed according to the timing of hospital presentation. Finally, we examined patients who presented with acute HF and survived till the end of follow-up with HFrEF as outcome.

Comparisons of outcomes between groups were made by 2-sided *P* value. To minimize concern about comparison of the treatment effect in subgroups, estimates were compared by test of interaction on the log scale (Supplemental Methods).^16^ A *P*-value <0.05 was taken to indicate that the difference between the effects in women and men was unlikely to have occurred simply by chance (Supplemental Methods).^16^ All statistical analyses were performed using R, version 3.4.4 (R Foundation for Statistical Computing, Vienna, Austria).

## Results

A total of 87,812 ACS patients met the inclusion criteria; of these patients 25,187 (28.7%) had acute HF on hospital admission. There were 30,922 (35.2%) women. There were 56,038 patients with STEMI (33.6% women) and 31,774 patients with NSTE-ACS (38.0% women) (Supplemental Figure 1). The proportion of patients categorized in each Killip class category is reported in Supplemental Figure 2. Cardiogenic shock (Killip class 4) was diagnosed in 5.0% of women and 3.3% of men with ACS. Women were also more likely to be in Killip class 2 (21.4% vs 17.4%) and in Killip class 3 (7.4% vs 5.2%).

### Baseline characteristics before inverse probability weighting

Baseline characteristics of the overall ACS population sorted by sex are shown in Supplemental Table 1. Many of the sex differences were not consistent between women and men, as reflected by the low standardized difference (<10%) for all variables. The only notable differences were that women were older than men, were more likely to have diabetes and hypertension, and were less frequently current smokers. Women received on average more medications for the management of HF, namely nitrates, diuretics, inotropes, and digoxin.

### Female sex and outcomes following acute coronary syndromes

**Figure 1:**
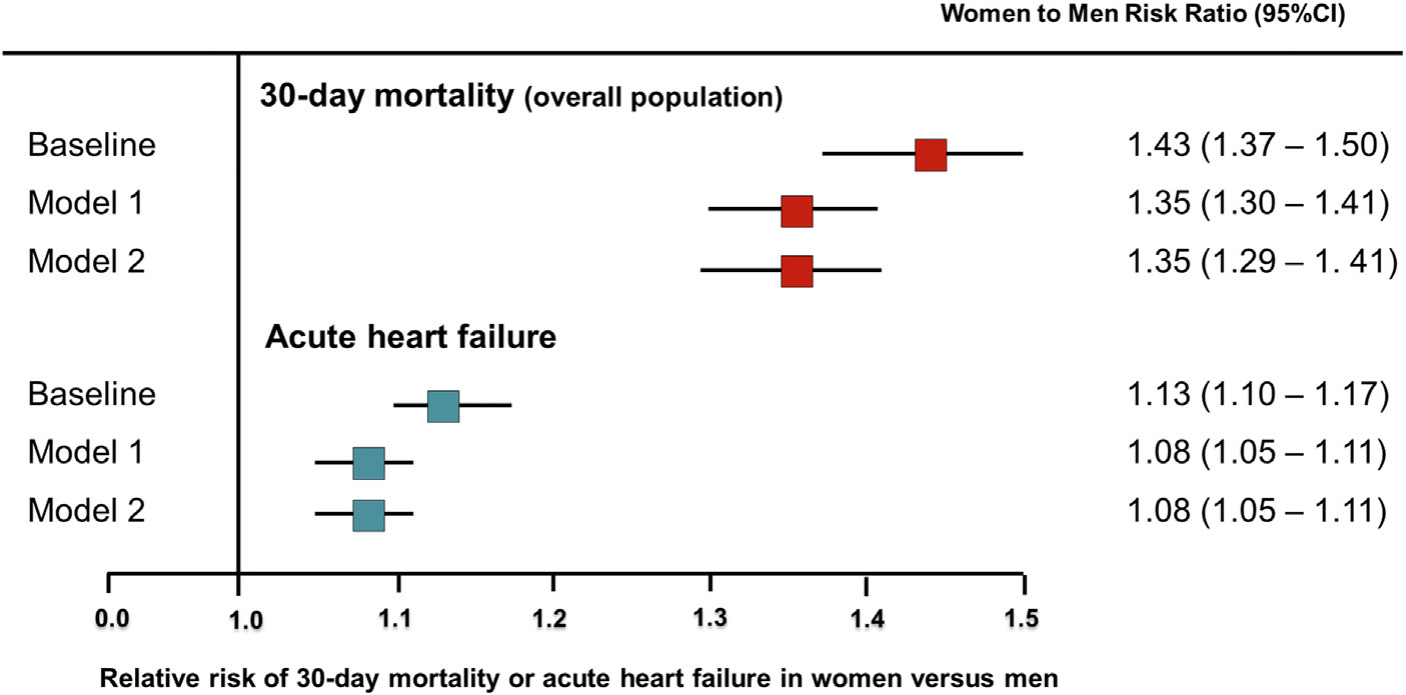
**Sequential Inverse Probability Weighting: Effect of Medications Use on the Risk of Heart Failure at Presentation or 30-Day Mortality in Patient With Acute Coronary Syndromes: Risk Ratios for Women vs Men** The following covariates are sequentially included in the adjusted models. Baseline: Adjusted model for baseline characteristics including age, cardiovascular risk factors, history of coronary heart disease, history of cardiovascular disease, and clinical characteristics (covariates reported in Table 1). Model 1: Adjusted baseline model, plus medications administered on admission (nitrates, inotropes, diuretics, digoxin, aspirin, clopidogrel, heparins, glycoprotein IIb/IIIa inhibitors). Model 2: Adjusted Model 1, plus medications administered during hospital stay (ACE inhibitors/Angiotensin receptor blockers, beta-blockers). ACE = angiotensin-converting enzyme.

Stabilized weighting using the inverse propensity score of being classified into a specific sex category resulted in achieving balance in baseline characteristics between women and men. Women with ACS (Table 1) were at increased risk of the primary outcome of 30-day mortality compared with men (absolute difference 3.5%; RR: 1.43; 95% CI: 1.37-1.50). Women were also at increased risk of developing acute HF at presentation (absolute difference 2.6%; RR: 1.13; 95% CI: 1.10-1.17). Adjustment for medications given on admission and during hospital stay yielded similar results (Figure 1).

### Female sex and outcomes stratified by acute coronary syndrome type

Mortality risk was higher in women compared with men with an absolute difference in 30-day mortality of 5.4% in those with STEMI (RR: 1.65; 95% CI: 1.56-1.73) and an absolute difference of 1.4% in those with NSTE-ACS (RR: 1.18; 95% CI: 1.09-1.28) (Table 2). Similarly, acute HF risk was higher in women with STEMI compared with NSTE-ACS. Women with STEMI had a 4.7% greater risk of acute HF compared with men (RR: 1.24; 95% CI: 1.20-1.29), whereas the risk of acute HF did not differ between women and men with NSTE-ACS (absolute difference 0.5%; RR: 1.02; 95% CI: 0.97-1.08). There were differences in estimates of 30-day mortality and acute HF in STEMI vs NSTE-ACS patients (*P*_interaction_ < 0.001). (Supplemental Tables 2 and 3).

**Table 2:** Clinical Factors and Outcomes Stratified by Sex and Acute Coronary Syndrome Type: Inverse Probability Weighting

Characteristics	STEMI			NSTE-ACS		
	Women (n = 18,855)	Men (n = 37,183)	Standardized Difference	Women (n = 12,067)	Men (n = 19,707)	Standardized Difference
Age (y)	62.7 ± 12.2	62.9 ± 11.8	−0.01	64.6 ± 11.7	64.8 ± 11.4	−0.01
Cardiovascular risk factors						
Family history of CAD	38.6	38.4	0.004	52.6	52.6	−0.001
Diabetes	24.5	24.7	−0.005	27.8	28.0	−0.005
Hypertension	65.2	65.8	−0.01	76.4	76.3	0.001
Hypercholesterolemia	46.1	46.6	−0.01	57.3	57.8	−0.01
Current smokers	45.0	44.6	0.01	38.7	38.4	0.01
Former smokers	1.7	1.7	−0.001	2.1	2.0	0.005
Clinical history of CHD						
Prior stable angina	20.9	20.8	0.0004	42.4	42.2	0.004
Prior myocardial infarction	15.6	15.2	0.01	26.0	25.7	0.005
Prior PCI	8.0	7.6	0.01	10.1	10.1	0.001
Prior CABG	2.7	2.2	0.03	6.4	6.3	0.005
Clinical history of CVD						
Peripheral artery disease	4.5	4.3	0.01	7.1	7.0	0.002
Prior stroke	4.5	4.5	0.0004	5.9	5.7	0.01
Clinical presentation on admission						
ST-segment shifts in anterior leads (at ECG)	42.6	42.2	0.01	44.9	45.1	−0.003
SBP at admission (mm Hg)	136.0 ± 29.5	136.5 ± 28.2	−0.02	138.6 ± 27.4	138.7 ± 27.0	−0.002
Heart rate at admission (beats/min)	82.9 ± 21.4	83.1 ± 21.0	−0.01	81.4 ± 21.6	81.4 ± 21.7	0.002
Serum creatinine at admission (mg/dL)	1.4 ± 0.9	1.2 ± 0.7	0.10	1.4 ± 1.4	1.3 ± 0.9	0.06

Outcomes			*P* Value			*P* Value
Primary outcome: 30-d mortality	15.3	9.9	<0.001	9.9	8.5	<0.001
Risk ratio (95% CI)	1.65 (1.56-1.73)		<0.001	1.18 (1.09-1.28)		<0.001
Secondary outcome: acute heart failure	33.7	29.0	<0.001	25.6	25.1	0.355
Risk ratio (95% CI)	1.24 (1.20-1.29)		<0.001	1.02 (0.97-1.08)		0.355

Values are mean ± SD or % unless otherwise indicated. SI conversion factor: To convert serum creatinine to mmol/L, multiply by 88.4.

CABG = coronary artery bypass graft; CAD = coronary artery disease; CHD = coronary heart disease; CVD = cardiovascular disorders; NSTE-ACS = non-ST-segment elevation acute coronary syndrome; PCI = percutaneous coronary intervention; SBP = systolic blood pressure; STEMI = ST-segment elevation myocardial infarction.

### Influence of reperfusion therapy on outcomes

Additional analysis was done in those patients with acute HF who underwent reperfusion therapy. Among STEMI patients, a substantial proportion, 41.5% of women and 29.4% of men, did not receive reperfusion therapy (Supplemental Figure 3). The mortality rate consistently decreased in both sexes but still remained higher in women than men (24.0% vs 20.1%; RR: 1.25; 95% CI: 1.12-1.39) (Table 3).

**Table 3:** Clinical Factors and 30-Day Mortality Stratified by Sex in STEMI Patients With Heart Failure on Admission Undergoing Reperfusion Therapy (Primary Percutaneous Coronary Intervention or Fibrinolysis): Inverse Probability Weighting

Characteristics	Women (n = 3,029)	Men (n = 5,491)	Standardized Difference
Age (y)	63.9 ± 11.9	64.4 ± 11.3	−0.04
Cardiovascular risk factors			
Family history of CAD	37.6	36.6	0.02
Diabetes	28.1	28.3	−0.004
Hypertension	69.6	69.7	−0.002
Hypercholesterolemia	46.4	47.4	−0.02
Current smokers	43.3	42.8	0.01
Former smokers	1.7	1.7	−0.004
Clinical history of CHD			
Prior stable angina	23.2	23.4	−0.005
Prior MI	15.8	16.6	−0.02
Prior PCI	9.0	7.9	0.04
Prior CABG	2.6	2.7	−0.006
Clinical history of CVD			
Peripheral arterial disease	5.0	5.1	−0.003
Prior stroke	5.2	5.3	−0.004
Clinical presentation on admission			
ST-segment shifts in anterior leads (at ECG)	52.8	51.1	0.03
SBP at admission, mm Hg	130.5 ± 29.5	130.9 ± 28.7	−0.01
Heart rate at admission, beats/min	87.1 ± 22.8	87.1 ± 22.4	−0.004
Serum creatinine levels at admission, mg/dL	1.4 ± 0.9	1.2 ± 0.7	−0.06

Outcomes			*P* Value
30-d mortality	24.0	20.1	<0.001
Risk ratio (95% CI)	1.25 (1.12-1.39)		<0.001

Values are mean ± SD or % unless otherwise indicated.

SI conversion factor: To convert serum creatinine to μmol/L, multiply by 88.4.

CABG = coronary artery bypass graft; CAD = coronary artery disease; CHD = coronary heart disease; CVD = cardiovascular disease; MI = myocardial infarction; PAD = peripheral artery disease; PCI = percutaneous coronary intervention; SBP = systolic blood pressure; STEMI = ST-segment elevation myocardial infarction.

### Influence of delay to hospital presentation on outcomes

**Figure 2:**
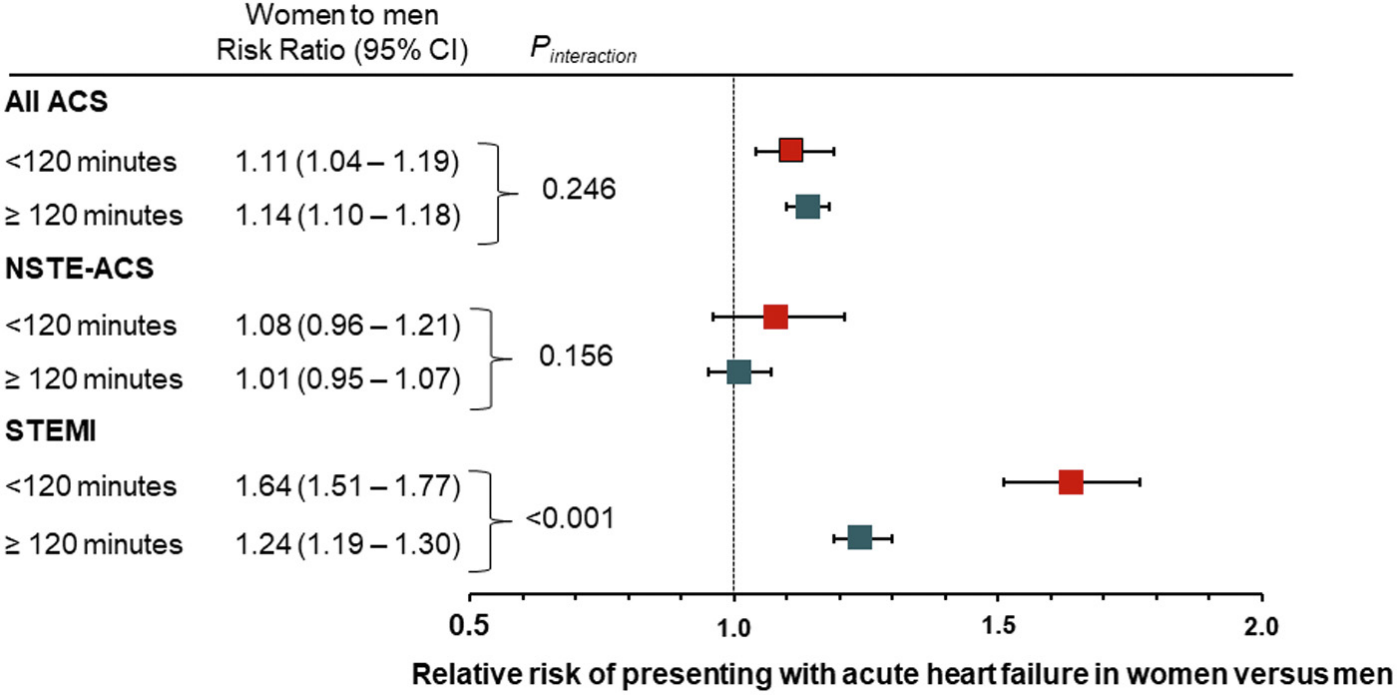
**Inverse Probability Weighting: Heart Failure Risk Stratified According to Time to Hospital Admission in Patients With Acute Coronary Syndromes: Risk Ratios for Women vs Men** Influence of delay to hospital presentation on outcomes. Outcomes in patients with time from symptom onset to hospital presentation ≥120 minutes vs <120 minutes were examined. Horizontal lines indicate corresponding 95% confidence intervals for the corresponding risk ratio. ACS = acute coronary syndrome; NSTE-ACS = non-ST-segment elevation acute coronary syndrome; STEMI = ST-segment elevation myocardial infarction.

Outcomes in patients with time from symptom onset to hospital presentation ≥120 minutes vs <120 minutes (Figure 2) were examined. Only 21.2% of ACS women presented within 120 minutes of symptom onset, but also 26.1% of men did so (Supplemental Table 4). Although acute HF increased with increasing time to hospital presentation in both women and men with ACS, we found a higher incidence of acute HF in women compared with men either when time from symptom onset to hospital presentation was more (≥120 minutes) or less (<120 minutes) delayed (RR: 1.14; 95% CI: 1.10-1.18 vs 1.11, 95% CI: 1.04-1.19; *P*_interaction_ = 0.245). The same pattern was seen in STEMI where sex differences in outcomes persisted for early (<120 minutes) and late (≥120 minutes) presentations (RR: 1.64; 95% CI: 1.51-1.77 and 1.24; 95% CI: 1.19-1.30, respectively; *P*_interaction_ < 0.001) (Supplemental Table 5). By contrast, there was no difference in acute HF development in NSTE-ACS by sex either in early (RR: 1.08; 95% CI: 0.96-1.21) or late (RR: 1.01; 95% CI: 0.95-1.07) presentation (*P*_interaction_ = 0.156) (Supplemental Table 6). The results of the interaction tests are reported in Supplemental Table 7.

### Female sex and mortality in acute heart failure

Among the overall ACS population, 10,442 women and 14,745 men had acute HF on hospital admission. Women and men were matched for baseline characteristics. Women were at increased risk of 30-day mortality compared with men (29.8% vs 25.5%; RR: 1.24; 95% CI: 1.17-1.31) (Supplemental Table 8). The mortality rates were attenuated in the cohort of ACS patients without clinical acute HF on presentation, nonetheless, the sex difference in 30-day mortality persisted (Supplemental Table 9).

### Associations between sex and Heart Failure types

Acute HF as measured by elevated Killip (≥2) class was not simply a surrogate marker for HFrEF (Supplemental Figure 4). The majority of ACS patients with acute HF showed relatively preserved (>40%) resting LV function at hospital discharge (Supplemental Table 10). In those with STEMI, women with acute HF were more likely to have HFrEF compared with men (RR: 1.12; 95% CI: 1.04-1.21). In contrast, in those with NSTE-ACS, women were less likely to have HFrEF than their male counterparts (RR: 0.73; 95% CI: 0.66-0.81; *P*_interaction_ < 0.001) (Supplemental Tables 11 and 12).

### Multivariable analysis of baseline clinical factors associated with outcomes

**Figure 3:**
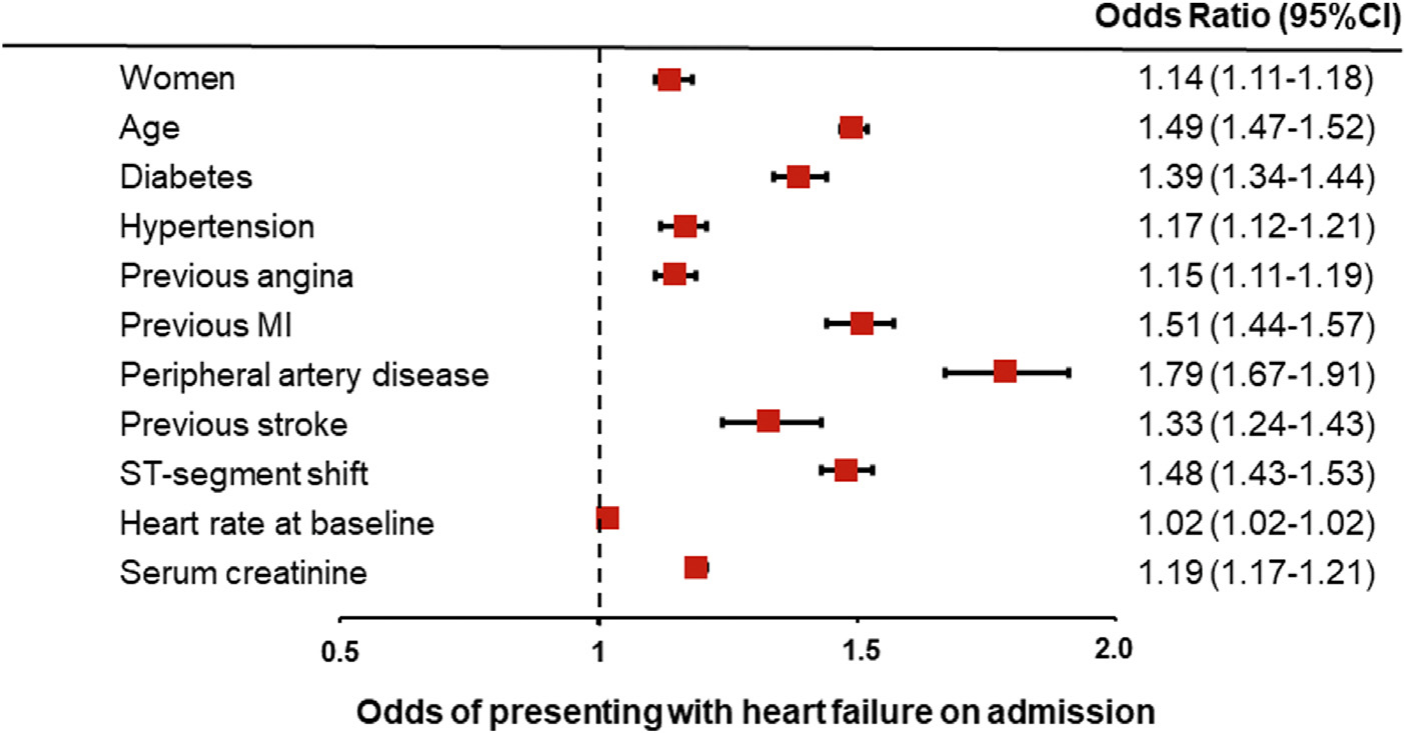
**Clinical Factors Associated With Development of Heart Failure in Patients With Acute Coronary Syndrome: Multivariable Analysis** Adjusted model for baseline characteristics including age, cardiovascular risk factors, history of coronary heart disease, history of cardiovascular disease, and clinical characteristics (covariates reported in Table 1). Horizontal lines indicate corresponding 95% confidence intervals for the corresponding risk ratios. MI = myocardial infarction.

**Figure 4:**
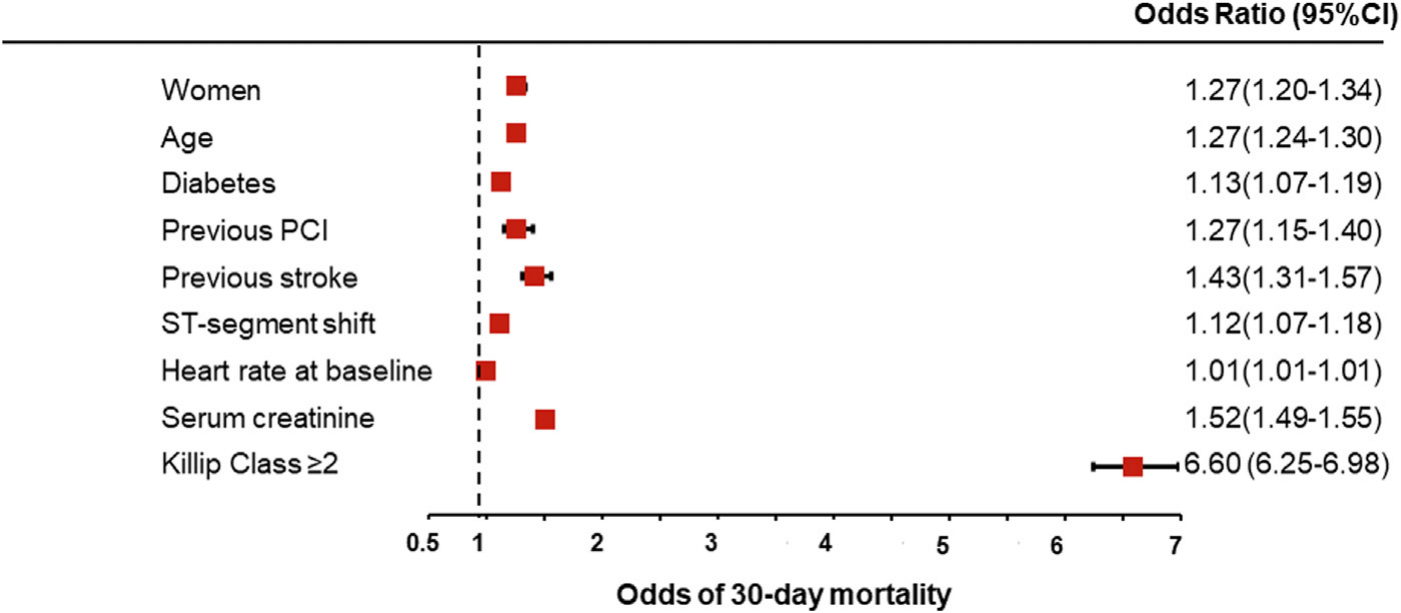
**Clinical Factors Associated With 30-Day Mortality in Patients With Acute Coronary Syndrome: Multivariable Analysis** Adjusted model for baseline characteristics including age, cardiovascular risk factors, history of coronary heart disease, history of cardiovascular disease, and clinical characteristics (covariates reported in Table 1). Horizontal lines indicate corresponding 95% confidence intervals for the corresponding odds ratios. PCI = percutaneous coronary intervention.

A multivariable model was created to further examine the effect of sex on acute HF (Figure 3) and 30-day mortality (Figure 4). Female sex was independently associated with acute HF (OR: 1.14; 95% CI: 1.11-1.18) and 30-day mortality (OR: 1.27; 95% CI: 1.20-1.34). Multivariable analysis also showed that acute HF was independently associated with 30-day mortality (OR: 6.60; 95% CI: 6.25-6.98).

## Discussion

Our study identified 4 key findings. First, women with ACS had a higher 30-day mortality when compared with men with ACS. The 30-day mortality risk was higher in women presenting with STEMI compared to those presenting with NSTE-ACS. Second, women were at increased risk of presenting with acute HF when compared with men, although this difference was only seen among the subset of patients with STEMI. Third, the presence of acute HF increased the risk of mortality for both sexes. Fourth, in those patients with STEMI, women with acute HF were more likely to have HFrEF compared with men (Central Illustration).

**Central Illustration:**
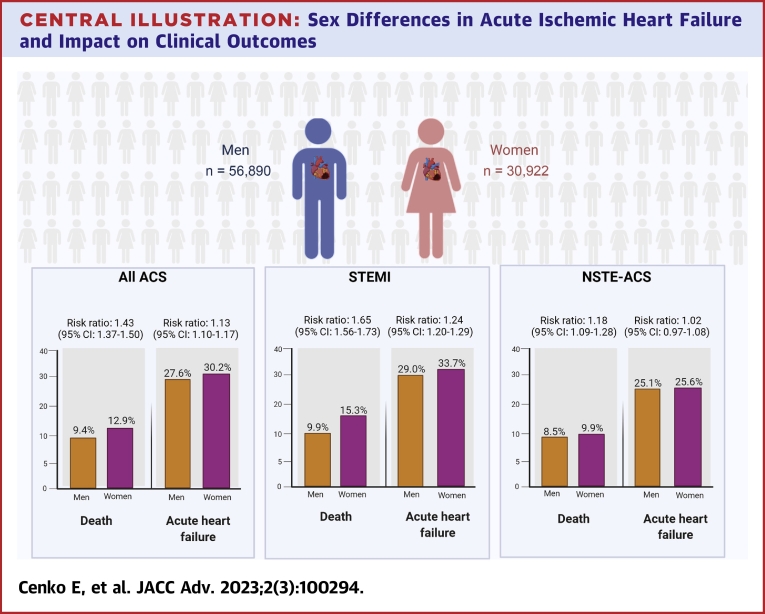
**Sex Differences in Acute Ischemic Heart Failure and Impact on Clinical Outcomes** The Central Illustration shows the absolute risk and risk ratios between women and men for 30-day mortality and acute heart failure, stratified by type of ACS presentation. Among the 56,890 men and 30,922 women included in the study, female patients had a higher 30-day mortality and acute heart failure risk, especially after STEMI. Figure created with BioRender.com.

### Sex differences in 30-day mortality after acute coronary syndromes

ACS is a unifying hierarchical term that subtends distinct subgroups of clinical presentations and related mortality risks. Although prior studies suggested that women were at increased risk of adverse outcomes after ACS^17^, more recent studies suggested that differences by sex only occur in patients presenting with STEMI.^7^^,^^18^ A meta-analysis, using data from 35 studies involving 18,555 women and 49,981 men with STEMI treated with primary PCI found that women had nearly 1.5 times the adjusted risk for in-hospital mortality compared with men.^4^ By contrast, data about the prognostic implication of sex in patients with NSTE-ACS are more contradictory. Another study that included patient data from 1,057 US hospitals encompassing a total of 361,429 patients from the National Registry of Myocardial Infarction (NRMI) found that the adjusted mortality was 15 to 20% higher in women than in men, regardless of type of myocardial infarction.^19^ In contrast, a study based on the National Inpatient Sample database found that women with NSTEMI had 10% lower odds of in-hospital mortality than men after accounting for differences in age, lower use of PCI, and comorbidities.^20^ Similar findings were also seen when previous studies have grouped patients without STEMI in the broader definition of NSTE-ACS. An analysis of the thrombolysis in myocardial infarction clinical trial database found that women had higher 30-day mortality compared with men in unadjusted models but a 16% lower risk of mortality after multivariable adjustment.^6^

In the current study, we used inverse probability weighting models to examine sex difference in 30-day mortality. The sex gap in mortality persisted among both STEMI and NSTE-ACS patients. Women with STEMI had a 65% increased risk of 30-day mortality as compared with men, and an 18% excess 30-day mortality in those with NSTE-ACS. Notably, the relative risks from these subgroups significantly differed from each other, as confirmed by the interaction test supporting the idea that there are more notable sex differences in the mortality in patients with STEMI compared with those of NSTE-ACS.

In our study, differences in mortality between men and women with NSTE-ACS were attenuated after adjustment for baseline characteristics. Because statistical models varied substantially between our study and previous work, it is difficult to determine whether the magnitude of the association between sex and mortality in NSTE-ACS that we identified is clinically significant. Perhaps, not every woman with NSTE-ACS has the same excess risk of cardiovascular mortality as compared with men. This is not surprising as the mechanisms behind the sex difference in mortality after ACS have not been identified in full.

### Sex differences in acute heart failure after acute coronary syndromes

Despite advances in the treatment of ACS, HF post-ACS remains frequent. While most studies have shown an increased risk of acute HF complicating acute myocardial infarction in women vs men, this issue remains controversial.^21^ In the NRMI study which included 606,500 cases of myocardial infarction from 1994 to 2000, women were more likely than men to develop acute HF at the time of hospital presentation.^22^ Similarly, in an Australian cohort of patients mainly constituted by acute myocardial infarction, women were more likely to develop acute HF during admission or within 28 days from the index event.^23^ In contrast, a study examining patients in Alberta, Canada found that women were less likely than men to develop acute HF during hospitalization for myocardial infarction.^24^ Reasons for divergent results may include lack of information on potential factors that may contribute to this sex difference including older age, increased cardiovascular risk profile, severity of clinical presentation, and differences in reperfusion time.^21^^,^^25^ Additionally, no direct statistical comparisons of the RRs for men and women were done in prior work. These questions form a basis for further investigations.

### Mechanism of the interaction between sex and acute heart failure

Our cohort included patients admitted with both STEMI and NSTE-ACS. Acute HF complicating STEMI was more common in women at 33.7% compared with 29.0% for men. In contrast, in NSTE-ACS patients, the risk for acute HF was similar between the sexes, 25.6% vs 25.1% in women and men, respectively. The mechanism by which female sex adversely affects HF risk post-ACS was investigated in our study. We found that the mechanism was not attributable to older age, comorbidities, or treatment as we weighted such variables in women vs men and created a sample in which outcomes were independent of measured baseline covariates including anterior ST-segment shift as a surrogate marker for infarct size. Our results did not support the hypothesis that women seek treatment later than men and therefore are more likely to develop HF or have worse outcomes. The incidence of acute HF in STEMI was persistently higher in women compared with men, regardless of the time to hospital presentation (35.5% vs 30.7% and 34.3% vs 24.2% in late and early presentations). In comparison, in NSTE-ACS patients, delay to hospital presentation did not significantly correlate with rates of acute HF among women and men. Our findings add to the literature suggesting a sex-based difference in myocardial vulnerability to ischemia such as that triggered by STEMI. The exact mechanism cannot be established in this study, but may include differences in impaired coronary microvascular flow and tissue perfusion in women compared with men.^26^

### Adjusting sex difference in mortality for heart failure

Sex differences in the risk of acute HF post-ACS is of particular importance as the development of HF has been associated with a markedly increased mortality risk. In the NRMI, in-hospital mortality was 24% for those with acute HF vs 6.2% for those without.^27^ Sex-specific comparisons were not performed. Under the assumption that acute HF is one of the mediators of the effects of ACS on mortality, we estimated the multivariable-adjusted effect of sex on acute HF and 30-day mortality. Female sex was independently associated with acute HF at presentation and 30-day mortality. Multivariable analysis showed that acute HF was independently associated with 30-day mortality. It follows that women are prone to develop acute HF, which is the most powerful predictor of death. However, female sex can be a predictor of death even independently of acute HF, thus additional mechanisms are likely important as well.

Other mechanisms for differences in outcomes have been explored. Previous studies have shown that there is excess bleeding risk in women; however, there are no data showing that reducing bleeding events improves outcomes.^28^ Women are also at higher risk of complications after coronary revascularization. In an angiographic analysis,^26^ suboptimal thrombolysis in myocardial infarction blood flow 0 to 2, despite minimum residual percent diameter stenosis<10% in women with STEMI, was higher than in men, even after adjustment for baseline differences including symptom-to-hospital presentation time. These findings highlight the ongoing need to accurately account for biologic factors specific to women with acute ischemia.

### Sex differences in the type of heart failure after acute coronary syndromes

Sex differences in LV dysfunction might contribute to an increased risk of acute HF complicating ACS in women compared with men. Previous studies have reported that a significant proportion of chronic HF cases have preserved LV systolic function. In the current study, among patients who developed acute HF, we found that only about 50% of patients with ACS had reduced EF. There were notable sex differences in LV systolic function in the patients with STEMI and NSTE-ACS. Women were more likely than men to have reduced LVEF after STEMI whereas they were less likely than men to have reduced LVEF after NSTE-ACS. This finding may explain some of the excess mortality and incidence of acute HF in women. This finding also reinforces the growing clamor for appropriate sex-specific analyses for questions such as response to drugs in acute HF therapies and risk stratification of ACS.

### Study limitations

Some limitations of our study should be acknowledged. As an observational study, we cannot completely exclude residual confounding due to unmeasured variables. Concern about bias in baseline measured characteristics, interventional strategies, and treatment of acute HF was minimized using a parametric balancing strategy by inverse propensity weighting based on the propensity score. Determination of Killip class reflects clinical practice and therefore is susceptible to differential interpretation. However, guidelines still recommend Killip classification as the best possible solution to categorize patients with acute HF on admission.^14^^,^^29^ Our data included hospitalized ACS patients only and did not account for out-of-hospital deaths. Still, such potential bias would probably affect men and women similarly and probably not explain the observed sex difference. Echocardiography was routinely performed at discharge as recommended by international guidelines.^30^ We, therefore, were unable to determine the relationship between HFrEF and 30-day mortality. Finally, results may not be definitive without replication.

## Conclusions

There are sex-based differences in 30-day mortality and acute HF presentation after ACS, which are independent of age, comorbidities, and delivery of care. Awareness of sex-related differences in ACS presentation and outcomes is an important consideration when personalizing risk stratification and treatment.

## Funding support and author disclosures

The authors have reported that they have no relationships relevant to the contents of this paper to disclose.

PERSPECTIVES **COMPETENCY IN PATIENT CARE AND PROCEDURAL SKILLS:** In patients presenting with ACS, there are important sex differences in 30-day mortality and risk for HF at presentation. Women have higher mortality and are more likely to present with HF when compared with men. Strategies to improve outcomes in the ACS population should include consideration of sex-specific differences.

**TRANSLATIONAL OUTLOOK:** There is limited understanding of the mechanisms responsible for sex-specific differences in presentation and outcomes in patients with ACS. Understanding the mechanisms responsible for sex differences is crucial in order to improve outcomes.
