# Supplementary material for: Sex Differences in Heart Failure Following Acute Coronary Syndromes
Source: JACC Adv. 2023 Apr 26;2(3):100294. doi: 10.1016/j.jacadv.2023.100294 (PMC11198630; doi:10.1016/j.jacadv.2023.100294)
Supplement: Supplementary data [file mmc1.docx]

**SUPPLEMENTAL APPENDIX**

**SUPPLEMENTAL METHODS**

**Comparison of the characteristics of the included and excluded populations**

| **Characteristics** | **Included**  **(N=87,812)** | **Removed**  **(N=38,547)** | **Standardized difference** | |
| --- | --- | --- | --- | --- |
| Age (years) | 63.6±11.7 | 63.8±11.5 | -0.0172 | |
| **Cardiovascular risk factors (overall)** | 81897 (93.3) | 32279 (83.7) | 0.3020 | |
| Family history of CAD | 38198 (43.5) | 10455 (27.1) | 0.3478 | |
| Diabetes | 22903 (26.1) | 10050 (26.1) | 0.0002 | |
| Hypertension | 61174 (69.7) | 25554 (66.3) | 0.0723 | |
| Hypercholesterolemia | 44576 (50.8) | 16794 (43.6) | 0.1445 | |
| Current smokers | 36965 (42.1) | 10774 (28.0) | 0.2998 | |
| Former smokers | 1582 (1.8) | 230 (0.6) | 0.1109 | |
| **Clinical history of CHD** | | | | |
| Prior stable angina | 25039 (28.5) | 10752 (27.9) | 0.0138 | |
| Prior myocardial infarction | 16650 (19.0) | 9703 (25.2) | -0.1502 | |
| Prior PCI | 7477 (8.5) | 2798 (7.3) | 0.0466 | |
| Prior CABG | 3218 (3.7) | 2241 (5.8) | -0.1013 | |
| **Clinical history of CVD** | | | | |
| Peripheral artery disease | 4596 (5.2) | 1948 (5.1) | 0.0082 | |
| Prior stroke | 4262 (4.9) | 2434 (6.3) | -0.0637 | |
| **Clinical presentation** | | | | |
| ST-segment shifts in anterior leads (at ECG) | 37918 (43.2) | 15099 (39.2) | 0.0816 | |
| Systolic blood pressure at baseline (mmHg) | 137.3±28.0 | 137.4±29.4 | -0.0036 | |
| Heart rate at baseline (bpm) | 82.5±21.3 | 84.8±19.8 | -0.1118 | |
| Serum creatinine at admission (mg/dL) | 1.2±0.8 | 1.3±1.0 | -0.0866 | |
| **Medications administered on admission** | | | | |
| Nitrates | 59499 (67.8) | 28417 (73.7) | -0.1314 | |
| Inotropes | 7765 (8.8 ) | 2938 (7.6 ) | 0.0444 | |
| Diuretics | 32287 (36.8 ) | 13957 (36.2 ) | 0.0116 |  |
| Digoxin | 6719 (7.7 ) | 2877 (7.5 ) | 0.0071 |  |
| Aspirin | 82384 (93.8 ) | 32692 (84.8 ) | 0.2947 |  |
| Clopidogrel | 62314 (71.0 ) | 24546 (63.7 ) | 0.1558 |  |
| Unfractionated heparin | 41355 (47.1 ) | 22219 (57.6 ) | -0.2124 |  |
| Glycoprotein IIb/IIIa inhibitors | 5902 (6.7 ) | 1702 (4.4 ) | 0.1007 |  |
| **In-hospital medications** | | | |  |
| ACE/ARBS | 59912 (68.2 ) | 23837 (61.8 ) | 0.1343 |  |
| Beta-blockers | 59295 (67.5 ) | 24001 (62.3 ) | 0.1104 |  |
| Data are mean ± standard deviation or n (%) unless otherwise specified.  Abbreviations: ACS= acute coronary syndrome; CABG =coronary artery bypass graft, CAD=coronary artery disease, CVD=cardiovascular disorders, IHD=ischemic heart disease, PCI=percutaneous coronary intervention | | | |  |

**Multiple Imputation using Chained Equation (MICE) algorithm**

Multiple Imputation using Chained Equation (MICE) algorithm is an efficient and popular method to fill in missing data where each missing value on some records is replaced by a value obtained from related cases in the whole set of records. Thus, imputation for clinical features was conducted using the chained equations across other features^1^. More specifically, MICE algorithm sequentially imputes the missing values of clinical features based on both observed values and previously imputed values. This sequential imputation is conducted via chained equations.

We tried multiple imputations using the MICE algorithm for the initial analyses to address the uncertainty in the imputation process. More specifically, we generated multiple imputed datasets and check whether the conclusions are consistent across the different imputed datasets. If the conclusions are consistent across multiple imputed datasets, we use a single imputed dataset (by MICE algorithm) as the final dataset to report the results of statistical analyses in the paper.

**Inverse probability weighting Analysis**

We used Inverse probability weighting (IPW) to balance the distribution of covariates between two patient groups. Note that we use Logistic Regression to estimate the propensity scores ({P}(Z=1 | x)) If *e* denotes the estimated propensity score (i.e. e=\hat{P}(Z=1 | x), where the patient x is included in patient group 1; then, 1-e = \hat{P}(Z=0 | x)), then the original sample is weighted by the following weights: Z/e+(1−Z)/ 1−e where Z represents the patient group. For instance, women (Z=1) are assigned a weight equal to the reciprocal of the propensity score (1/e), while men (Z=0) are assigned a weight equal to the reciprocal of one minus the propensity score (1/1-e). The weighting procedure for each sample balances the covariate distributions between two patient groups^2^.

Inverse probability of treatment weighting method can potentially result in unstable and biased estimates if some of the weights are very high. To avoid excessive weights, we compared results with other methods for handling confounding. We included probability of treatment variables in a multivariable model. We also used XGBoost, a decision-tree-based ensemble machine learning algorithm, as an alternative multivariable model for estimating the probability of treatment. Conclusions from theses analyses were the same as our current results. Further, we created a threshold for weights to avoid the impacts of the outliers (we use 0.01 as threshold). Therefore, the inverse probability of treatment weighting analyses presented in the current analysis were quite stable.

**Computation of Relative Risk and its Confidence Interval**

In a two-group cohort study, the risk ratio (RR, also called relative risk), is usually applied to compare risks of a health event between two independent binomial populations that differ by a demographic characteristic (i.e. sex, age) or by the level of exposure to a specific drug or risk factor. In such types of studies, data can be summarized in a confusion matrix as follows:

|  | **Risk of Designated Outcome** | |  |
| --- | --- | --- | --- |
|  | **Yes** | **No** | **Total** |
| **Exposed** | a | b | a+b (*H_1_*) |
| **Unexposed** | c | d | c+d (*H_0_*) |
| **Total** | a+c | b+c |  |

Where *H_1_* and *H_0_* correspond to the total number of exposed and unexposed patients, respectively, whereas *a* and *c* represent the number of exposed and unexposed patients at risk for the designated outcome, respectively.

RR will is defined as the ratio between the risk of outcome in exposed patients (*H_1_*) and the risk of outcome in unexposed patients (*H_0,_*) which can be summarized as:

$$RR=\frac{\left( \frac{a}{H_{1}} \right)}{\left( \frac{c}{H_{0}} \right)}$$

When applying this equation to an IPTW balanced population, $\frac{a}{H_{1}}$ will be assigned a weight equal to the reciprocal of the propensity score ($\frac{1}{e}$) and $\frac{c}{H_{0}}$ will be weighted by the reciprocal of one minus the propensity score ($\frac{1}{(1-e)}$).

In order to compute the lower and upper (1-α) confidence limit RR_L_ for RR, we operate in the assumption of log normal distribution. In particular, the variate $\log\frac{\left( \frac{a}{H_{1}} \right)}{\left( \frac{c}{H_{0}} \right)}$= $\log\frac{a}{H_{1}}- \log\frac{c}{H_{0}}$is approximately normally distributed with approximate mean log(RR) and estimated variance $\frac{1-\left( \frac{a}{H_{1}} \right)}{a}$ + $\frac{1-(\frac{c}{H_{0}})}{c}$ .

It follows that RR_L_  can be computed by solving the following equation:

$$\frac{\left[ log( \frac{\frac{a}{H_{1}}}{\frac{c}{H_{0}}})- \log({RR}_{L}) \right]}{\left[ \frac{1-\left( \frac{a}{H_{1}} \right)}{a} + \frac{1-(\frac{c}{H_{0}})}{c} \right]^{1/2}}=z_{1-\alpha}$$

Where $z_{1-\alpha}$, is the 100(1-α) percentage point of the N(O, 1) distribution

## **Comparison of means and prevalences in the weighted sample**

To evaluate the balance of the baseline covariate distributions between treatment and control groups, standardized difference (SD) is widely used in inverse probability of treatment weighting (IPTW) framework. For the baseline analysis, we use standard SD which is defined as follows: $\frac{m_{t}-m_{c}}{\sqrt{\frac{s_{t}^{2}+s_{c}^{2}}{2}}}$ for continuous variables and $\frac{m_{t}-m_{c}}{\sqrt{\frac{m_{t}(1-m_{t})+m_{c}(1-m_{c})}{2}}}$ for binary variable where $m_{t}, m_{c}$ are sample mean of the variables for treatment and control group, and $s_{t}^{2}, s_{c}^{2}$ are sample variance of the variables for treatment and control group, respectively. For IPTW analysis, we use weighted SD where $m_{t}, m_{c}$ are replaced to weighted sample mean of the variables for treatment and control group, and $s_{t}^{2}, s_{c}^{2}$ are replaced to weighted sample variance of the variables for treatment and control group, respectively. Weights are determined by the inverse probability of treatment received. In general, 0.1 is the reasonable threshold to determine whether two distributions are balanced (i.e., if SD > 0.1, the baseline covariate is imbalanced).^3^

**Interaction test**

The comparison of two estimated quantities, each with its standard error, is a general method that can be applied widely. We compared the risk ratios of 30-day mortality and acute heart failure from two subgroups (women versus men) sorted by ACS type (STEMI vs NSTE-ACS). These measures were always analyzed on the log scale because the distributions of the log ratios tend to be those closer to normal than of the ratios themselves. If the estimates are *E*1 and *E*2 with standard errors SE(*E*1) and SE(*E*2), then the difference *d*=*E*1 - *E*2 has standard error SE(*d*)=Ö[SE(*E*1)2 + SE(*E*2)2] i.e., the square root of the sum of the squares of the separate standard errors. The ratio *z*=*d*/SE(*d*) gives a test of the null hypothesis that in the population the difference *d* is zero, by comparing the value of *z* to the standard normal distribution. The 95% confidence interval (CI) for the difference is *d*-1.96SE(*d*) to *d*+1.96SE(*d*) ^4^.

**SUPPLEMENTAL RESULTS**

**Interaction test**

We tested **(Supplemental Table 2)** whether there is a significant interaction between sex (women versus men) and ACS type (STEMI versus NSTE-ACS) in function of the outcome (30-day mortality)**.** We obtained the logs of the risk ratios and their confidence intervals (rows 2 and 4). As 95% confidence intervals were obtained as 1.96 standard errors (SE) either side of the estimate, the SE of each log relative risk was obtained by dividing the width of its confidence interval by 2×1.96 (row 6). The estimated difference in log relative risks was *d*=E1- E2= 0.3353 (row 7) and its standard error 0.0488 (row 8). From these two values, we tested the interaction and estimated the ratio of the relative risks (with confidence interval). The test of interaction was the ratio of *d* to its standard error: z= 6.8709, which gave a P value <0.0001 when we referred it to a table of the normal distribution (row 10). The estimated interaction effect was exp =1.3984 (row 11). The confidence interval for this effect was 0.2397 to 0.4309 on the log scale (row 9). Transforming back to the relative risk scale, we got 1.2709 to 1.5386 (row 12). We repeated the analysis in function of the rates of acute heart failure on admission **(Supplemental Table 3),** and according to delay to hospital presentation across ACS groups **(Supplemental Table 7**). We also tested interaction in function of left ventricular ejection fraction≤40% in patients with acute heart failure on admission **(Supplemental Table 12) .** There was good evidence to support different estimates for 30-day mortality and acute heart failure on hospital admission in STEMI versus NSTE-ACS patients.

## **Supplemental Figure 1.** Flow Diagram


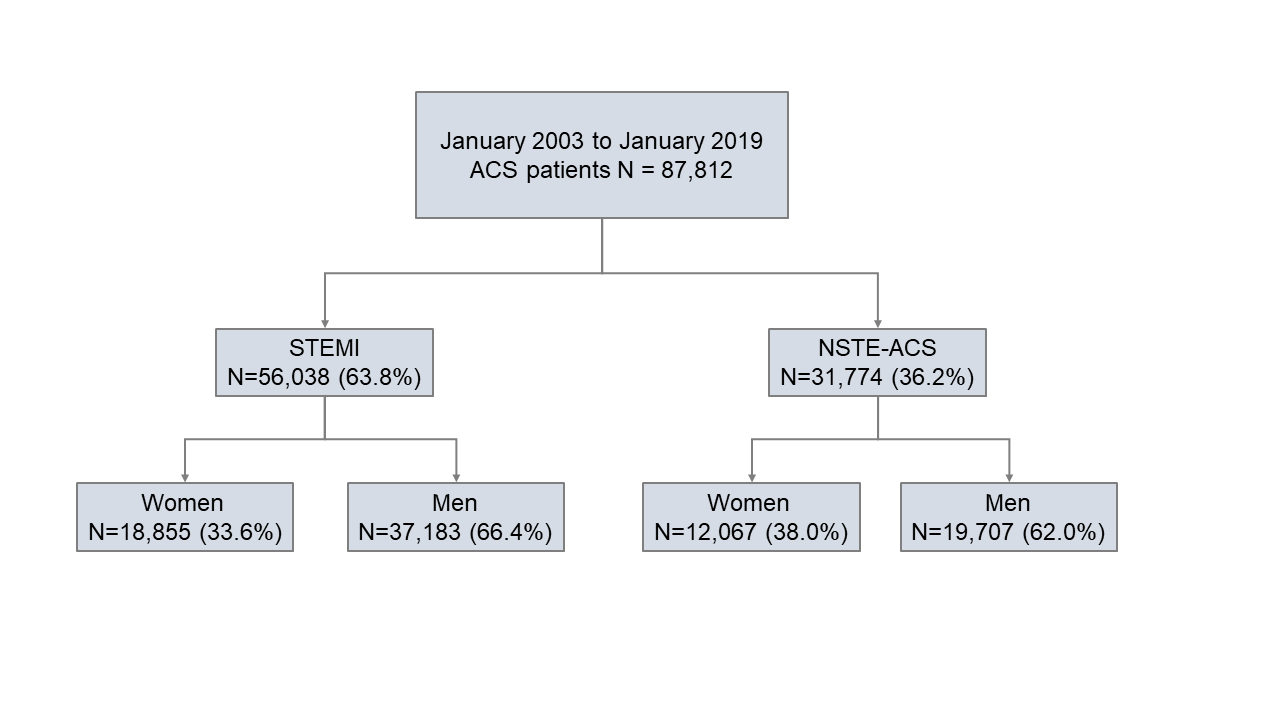


Abbreviations: ACS, acute coronary syndromes; NSTE-ACS, non-ST-segment elevation acute coronary syndromes; STEMI, ST-segment elevation myocardial infarction.

## **Supplemental Figure 2.** Killip classes sorted by sex and ACS type

**
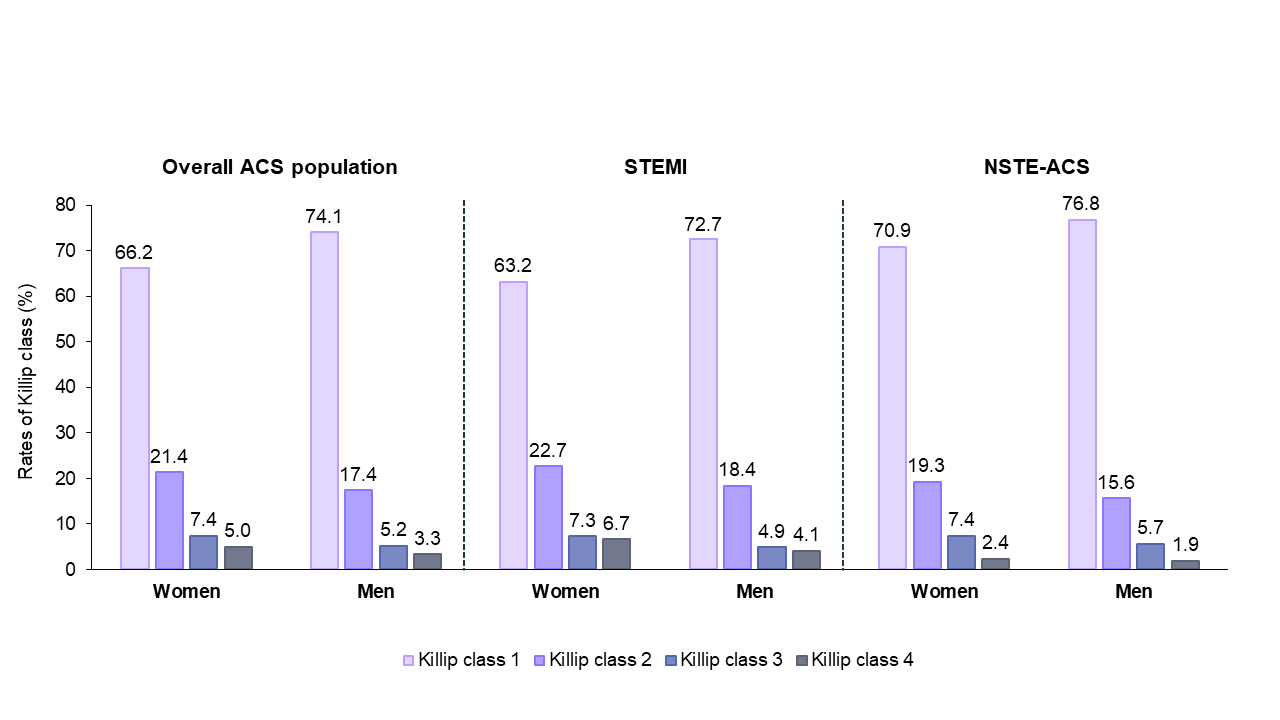
**

Abbreviations: ACS, acute coronary syndrome; NSTE-ACS, non-ST-segment elevation acute coronary syndrome; STEMI, ST-segment elevation myocardial infarction.

## **Supplemental Figure 3.** Rates of reperfusion therapy sorted by sex and ACS type

Abbreviations: ACS, Acute coronary syndromes; NSTE-ACS, Non –ST elevation acute coronary syndromes; STEMI, ST-elevation myocardial infarction

## **Supplemental Figure 4.** Heart failure with reduced ejection fraction in acute coronary syndromes

**
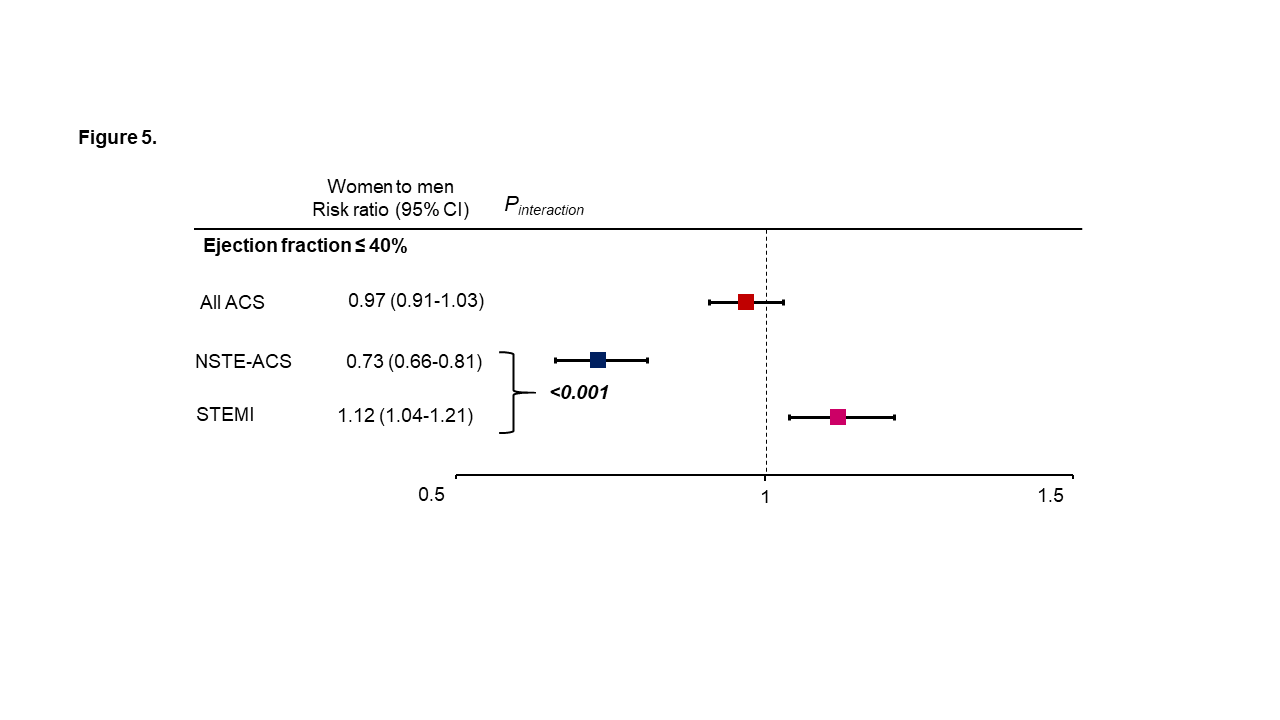
**

Abbreviations: ACS, Acute coronary syndromes; NSTE-ACS, Non –ST elevation acute coronary syndromes; STEMI, ST-elevation myocardial infarction

| **Supplemental Table 1.** Baseline characteristics of the overall population with ACS | | | |
| --- | --- | --- | --- |
| **Characteristics** | **Women**  **(N=30,922)** | **Men**  **(N=56,890)** | **Standardized difference** |
| Age (years) | 67.1 ± 11.0 | 61.7 ± 11.6 | 0.48 * |
| **Cardiovascular risk factors (overall)** |  |  |  |
| Family history of CAD | 14,024 (45.4) | 24,174 (42.5) | 0.06 * |
| Diabetes | 9,845 (31.8) | 13,058 (23.0) | 0.20 * |
| Hypertension | 23,887 (77.2) | 37,287 (65.5) | 0.26 * |
| Hypercholesterolemia | 16,642 (53.8) | 27,934 (49.1) | 0.09 * |
| Current smokers | 8,967 (29.0) | 27,998 (49.2) | -0.42* |
| Former smokers | 246 (0.8) | 1,336 (2.3) | -0.13 * |
| **Clinical history of CHD** |  |  |  |
| Prior stable angina | 9,991 (32.3) | 15,048 (26.5) | 0.13 * |
| Prior myocardial infarction | 5,214 (16.9) | 11,436 (20.1) | -0.08* |
| Prior PCI | 2,214 (7.2) | 5,263 (9.3) | -0.08* |
| Prior CABG | 738 (2.4) | 2,480 (4.4) | -0.11* |
| **Clinical history of CVD** |  |  |  |
| Peripheral artery disease | 1,685 (5.4) | 2,911 (5.1) | 0.015 |
| Prior stroke | 1,840 (6.0) | 2,422 (4.3) | 0.08 * |
| **Clinical presentation** |  |  |  |
| ST-segment shifts in anterior leads (at ECG) | 13,846 (44.8) | 24,072 (42.3) | 0.05* |
| SBP at admission (mmHg) | 137.8 ± 28.7 | 137.0 ± 27.7 | 0.03* |
| HR at admission (bpm) | 83.8 ± 22.3 | 81.8 ± 20.7 | 0.09 * |
| Serum creatinine at admission (mg/dL) | 1.1 ± 0.8 | 1.2 ± 0.9 | -0.12 * |
| Time to hospital presentation <120 minutes | 6,542 (21.2) | 14,848 (26.1) | -0.12 * |
| **Medications administered on admission** | | | |
| Nitrates | 21,280 (68.8) | 38,219 (67.2) | 0.04 * |
| Inotropes | 3,154 (10.2) | 4,611 (8.1) | 0.07 * |
| Diuretics | 12,942 (41.9) | 19,345 (34.0) | 0.16 * |
| Digoxin | 2,845 (9.2) | 3,874 (6.8) | 0.09 * |
| Aspirin | 28,794 (93.1) | 53,590 (94.2) | -0.04 * |
| Clopidogrel | 20,923 (67.7) | 4,1391 (72.8) | -0.11 * |
| Heparins | 14,112 (45.6) | 2,7243 (47.9) | -0.05* |
| Glycoprotein IIb/IIIa inhibitors | 2,026 (6.6) | 3,876 (6.8) | -0.01 * |
| **In-hospital medications** |  |  |  |
| ACE/ARBS | 21,405 (69.2) | 38,507 (67.7) | 0.03 |
| Betablockers | 20,776 (67.2) | 38,519 (67.7) | -0.01 |
| Data are presented as numbers (%) ore mean ± SD, unless otherwise specified.  Abbreviations: ACS, acute coronary syndromes; ACE, angiotensin converting enzyme; ARBs; angiotensin receptor blocker; CABG, coronary artery bypass graft; CAD, coronary artery disease; CHD, coronary heart disease; CVD, cardiovascular disorders; HR, heart rate; PCI, percutaneous coronary intervention; SBP, systolic blood pressure  *Indicates a –p-value<0.001 | | | |

| **Supplemental Table 2.** Interaction test: calculations for comparing two estimated risk ratios for 30-day mortality (women vs men) by Inverse probability weighting: STEMI vs NSTE-ACS. | | | |
| --- | --- | --- | --- |
|  |  | **Group 1**  **[STEMI]**  **(N = 56,038)** | **Group 2**  **[NSTE-ACS]**  **(N = 31,774)** |
| **1** | **RR** | 1.65 | 1.18 |
| **2** | **log RR** | 0.5008 | 0.1655 |
| **3** | **95% CI for RR** | 1.56 – 1.73 | 1.09 – 1.28 |
| **4** | **95% CI for log RR** | 0.4447 – 0.5481 | 0.0862 – 0.2469 |
| **5** | **Width of CI** | 0.1034 | 0.1607 |
| **6** | **SE (=width / (2*1.96))** | 0.0264 | 0.0410 |
| **Difference between log risk ratios** | | | |
| **7** | **d (=)** | **0.3353** | |
| **8** | **SE (d)** | **0.0488** | |
| **9** | **CI (d)** | **0.2397 – 0.4309** | |
| **10** | **Test of Interaction** | **6.8773 (p-value: <0.0001)** | |
| **Ratio of risk ratios** | | | |
| **11** | **RRR ( =exp(d) )** | 1.3984 | |
| **12** | **CI (RRR)** | 1.2709 – 1.5386 | |

| **Supplemental Table 3.** Interaction test: calculations for comparing two estimated risk ratios for acute heart failure on admission (women vs men) by Inverse probability weighting: STEMI vs NSTE-ACS | | | |
| --- | --- | --- | --- |
|  |  | **Group 1**  **[STEMI]**  **(N = 56,038)** | **Group 2**  **[NSTE-ACS]**  **(N = 31,774)** |
| **1** | **RR** | 1.24 | 1.02 |
| **2** | **log RR** | 0.2151 | 0.0198 |
| **3** | **95% CI for RR** | 1.20 – 1.29 | 0.97 – 1.08 |
| **4** | **95%CI for log RR** | 0.1823–0.2546 | -0.0305 – 0.0770 |
| **5** | **Width of CI** | 0.07232 | 0.1074 |
| **6** | **SE (=width / (2*1.96))** | 0.0184 | 0.0274 |
| **Difference between log risk ratios** | | | |
| **7** | **d (=)** | **0.1953** | |
| **8** | **SE (d)** | **0.0330** | |
| **9** | **CI (d)** | **0.1306 – 0.2601** | |
| **10** | **Test of Interaction** | **5.9122 (p-value <0.0001)** | |
| **Ratio of risk ratios** | | | |
| **11** | **RRR ( =exp(d) )** | 1.2157 | |
| **12** | **CI (RRR)** | 1.1395 – 1.2970 | |

| **Supplemental Table 4.** Inverse probability weighting: clinical factors and rates of heart failure on admission sorted by sex and delay to hospital presentation in ACS patients | | | | | | |
| --- | --- | --- | --- | --- | --- | --- |
|  | **Delay to hospital presentation**  **(<120 minutes)** | | | **Delay to hospital presentation**  **(**≥**120 minutes)** | | |
| **Characteristics** | **Women**  **(N=6,542)** | **Men**  **(N=14,848)** | **Standardized difference** | **Women**  **(N=24,380)** | **Men**  **(N=42,042)** | **Standardized difference** |
| Age (years) | 61.2 ± 12.2 | 61.3 ± 11.7 | -0.0063 | 64.1 ± 11.9 | 64.3 ± 11.6 | -0.0182 |
| **Cardiovascular risk factors** |  |  |  |  |  |  |
| Family history of CAD | 48.0 | 48.3 | -0.0062 | 42.4 | 42.0 | 0.0066 |
| Diabetes | 22.4 | 22.7 | -0.0085 | 26.7 | 26.9 | -0.0046 |
| Hypertension | 67.0 | 68.0 | -0.0209 | 70.1 | 70.1 | -0.0012 |
| Hypercholesterolemia | 53.2 | 54.2 | -0.0201 | 49.3 | 49.5 | -0.0049 |
| Current smokers | 49.4 | 49.6 | -0.0035 | 40.5 | 40.0 | 0.0101 |
| Former smokers | 1.3 | 1.4 | -0.0084 | 2.1 | 1.9 | 0.0073 |
| **Clinical history of CHD** |  |  |  |  |  |  |
| Prior stable angina | 27.6 | 27.5 | 0.0024 | 29.0 | 28.9 | 0.0020 |
| Prior myocardial infarction | 21.5 | 20.9 | 0.0162 | 18.8 | 18.4 | 0.0083 |
| Prior PCI | 9.1 | 8.4 | 0.0242 | 8.7 | 8.6 | 0.0037 |
| Prior CABG | 4.1 | 3.9 | 0.0106 | 3.9 | 3.6 | 0.0160 |
| **Clinical history of CVD** |  |  |  |  |  |  |
| Peripheral artery disease | 4.9 | 4.7 | 0.0054 | 5.5 | 5.4 | 0.0035 |
| Prior stroke | 4.3 | 4.2 | 0.0018 | 5.2 | 5.2 | 0.0033 |
| **Clinical presentation on admission** | |  |  |  |  |  |
| ST-segment shifts in anterior leads (at ECG) | 43.6 | 43.4 | 0.0032 | 43.4 | 43.2 | 0.0044 |
| SBP at admission (mmHg) | 137.4 ± 29.6 | 138. 0± 28.0 | -0.0197 | 136.9 ± 28.6 | 137.0 ± 27.6 | -0.0034 |
| HR at admission (bpm) | 80.8 ± 21.0 | 81.2 ± 21.2 | -0.0175 | 82.8 ± 21.8 | 82.8 ± 21.1 | 0.0002 |
| Serum creatinine at admission (mg/dL) | 1.4 ± 1.6 | 1.2 ± 1.0 | 0.0910 | 1.3 ± 1.4 | 1.2 ± 1.0 | 0.0550 |
| **Outcome** |  |  | ***P* value** |  |  | ***P* value** |
| Acute heart failure | 25.9 | 23.9 | 0.0024 | 31.7 | 28.9 | <0.0001 |
| Risk Ratio (95% CI) | 1.11(1.04 – 1.19) | | 0.0022 | 1.14 (1.10 – 1.18) | | <0.0001 |
| Data are presented as percentages (%) or mean ± standard deviation, unless otherwise specified.  Abbreviations: ACS, acute coronary syndromes; CABG, coronary artery bypass graft; CAD, coronary artery disease; CHD, coronary heart disease; CVD, cardiovascular disorders; HR, heart rate; PCI, percutaneous coronary intervention; SBP, systolic blood pressure | | | | | | |

| **Supplemental Table 5.** Inverse probability weighting: clinical factors and rates of heart failure on admission sorted by sex and delay to hospital presentation in STEMI patients | | | | | | |
| --- | --- | --- | --- | --- | --- | --- |
|  | **Delay to hospital presentation**  **(<120 minutes)** | | | **Delay to hospital presentation**  **(**≥**120 minutes)** | | |
| **Characteristics** | **Women**  **(N=4,065)** | **Men**  **(N=10,247)** | **Standardized difference** | **Women**  **(N=14,790)** | **Men**  **(N=26,936)** | **Standardized difference** |
| Age (years) | 62.4 ± 12.4 | 60.3 ± 11.7 | 0.1734 | 63.5 ± 12.1 | 63.8 ± 11.7 | -0.0236 |
| **Cardiovascular risk factors** |  |  |  |  |  |  |
| Family history of CAD | 39.9 | 44.4 | -0.0924 | 36.9 | 36.3 | 0.0119 |
| Diabetes | 18.3 | 21.1 | -0.0708 | 25.9 | 26.0 | -0.0015 |
| Hypertension | 57.1 | 63.4 | -0.1291 | 66.4 | 66.6 | -0.0037 |
| Hypercholesterolemia | 44.9 | 50.6 | -0.1143 | 45.0 | 45.3 | -0.0046 |
| Current smokers | 46.4 | 51.9 | -0.1102 | 42.8 | 42.0 | 0.0161 |
| Former smokers | 1.0 | 1.5 | -0.0460 | 1.8 | 1.8 | 0.0056 |
| **Clinical history of CHD** |  |  |  |  |  |  |
| Prior stable angina | 20.8 | 19.5 | 0.0329 | 21.2 | 21.3 | -0.0017 |
| Prior myocardial infarction | 21.1 | 16.7 | 0.1111 | 14.9 | 14.7 | 0.0068 |
| Prior PCI | 10.8 | 7.9 | 0.0977 | 7.8 | 7.5 | 0.0104 |
| Prior CABG | 2.5 | 2.5 | 0.0040 | 2.6 | 2.2 | 0.0265 |
| **Clinical history of CVD** |  |  |  |  |  |  |
| Peripheral artery disease | 3.6 | 3.9 | -0.0155 | 4.6 | 4.4 | 0.0088 |
| Prior stroke | 2.9 | 3.4 | -0.0281 | 4.9 | 4.9 | 0.0011 |
| **Clinical presentation on admission** |  |  |  |  |  |  |
| ST-segment shifts in anterior leads (at ECG) | 42.2 | 41.9 | 0.0064 | 42.7 | 42.3 | 0.0074 |
| SBP at admission (mmHg) | 131.2 ± 33.7 | 136.4 ± 28.2 | -0.1697 | 136.3±29.3 | 136.5 ± 28.1 | -0.0048 |
| HR at admission (bpm) | 78.5 ± 20.5 | 80.8 ± 20.7 | -0.1122 | 83.8 ± 21.6 | 83.8 ± 21.1 | -0.0035 |
| Serum creatinine at admission (mg/dL) | 1.3 ± 0.8 | 1.1 ± 0.6 | 0.0731 | 1.4 ± 1.0 | 1.1 ± 0.7 | 0.0806 |
| **Outcome** |  |  | ***P* value** |  |  | ***P* value** |
| Acute heart failure | 34.3 | 24.2 | <0.0001 | 35.5 | 30.7 | <0.0001 |
| Risk Ratio (95% CI) | 1.64 (1.51 – 1.77) | | <0.0001 | 1.24 (1.19 – 1.30) | | <0.0001 |
| Data are presented as percentages (%) or mean ±standard deviation, unless otherwise specified.  Abbreviations: CABG, coronary artery bypass graft; CAD, coronary artery disease; CHD, coronary heart disease; CVD, cardiovascular disorders; HR, heart rate; PCI, percutaneous coronary intervention; SBP, systolic blood pressure; STEMI, ST-segment elevation myocardial infarction. | | | | | | |

| **Supplemental Table 6.** Inverse probability weighting: clinical factors and rates of heart failure on admission sorted by sex and delay to hospital presentation in NSTE-ACS patients | | | | | | |
| --- | --- | --- | --- | --- | --- | --- |
|  | **Delay to hospital presentation**  **(<120 minutes)** | | | **Delay to hospital presentation**  **(**≥**120 minutes)** | | |
| **Characteristics** | **Women**  **(N=2,477)** | **Men**  **(N=4,601)** | **Standardized difference** | **Women**  **(N=9,590)** | **Men**  **(N=15,106)** | **Standardized difference** |
| Age (years) | 63.1 ± 11.9 | 63.4 ± 11.3 | -0.0231 | 65.1 ± 11.6 | 65.2 ± 11.3 | -0.0108 |
| **Cardiovascular risk factors** |  |  |  |  |  |  |
| Family history of CAD | 55.9 | 56.0 | -0.0023 | 51.6 | 51.6 | -0.0012 |
| Diabetes | 26.0 | 26.1 | -0.0009 | 28.3 | 28.5 | -0.0055 |
| Hypertension | 76.9 | 77.2 | -0.0085 | 76.2 | 76.1 | 0.0042 |
| Hypercholesterolemia | 60.8 | 61.4 | -0.0122 | 56.4 | 56.7 | -0.0077 |
| Current smokers | 45.1 | 44.8 | 0.0062 | 36.8 | 36.5 | 0.0057 |
| Former smokers | 1.3 | 1.2 | 0.0063 | 2.3 | 2.3 | 0.0052 |
| **Clinical history of CHD** |  |  |  |  |  |  |
| Prior stable angina | 43.7 | 43.6 | 0.0014 | 42.0 | 41.8 | 0.0044 |
| Prior myocardial infarction | 29.2 | 29.3 | -0.0019 | 25.0 | 24.7 | 0.0061 |
| Prior PCI | 9.8 | 9.3 | 0.0171 | 10.2 | 10.3 | -0.0040 |
| Prior CABG | 6.8 | 6.9 | -0.0053 | 6.3 | 6.1 | 0.0082 |
| **Clinical history of CVD** |  |  |  |  |  |  |
| Peripheral artery disease | 6.5 | 6.5 | 0.0035 | 7.2 | 7.2 | 0.0021 |
| Prior stroke | 6.0 | 5.8 | 0.0083 | 5.8 | 5.7 | 0.0082 |
| **Clinical presentation on admission** |  |  |  |  |  |  |
| ST-segment shifts in anterior leads (at ECG) | 46.3 | 46.6 | -0.0059 | 44.5 | 44.6 | -0.0022 |
| SBP at admission (mmHg) | 141.1 ± 28.0 | 141.1 ± 27.4 | -0.0012 | 137.9 ± 27.2 | 138.0 ± 26.8 | -0.0020 |
| HR at admission (bpm) | 81.9 ± 21.7 | 82.0 ± 22.9 | -0.0079 | 81.3 ± 21.5 | 81.1 ± 21.2 | 0.0067 |
| Serum creatinine at admission (mg/dL) | 1.3 ± 1.3 | 1.3 ± 0.9 | 0.0419 | 1.4 ± 1.4 | 1.3 ± 1.0 | 0.0465 |
| **Outcome** |  |  | ***P* value** |  |  | ***P* value** |
| Acute heart failure | 24.4 | 23.1 | 0.2147 | 25.9 | 25.7 | 0.7235 |
| Risk Ratios (95% CI) | 1.08 (0.96 – 1.21) | | 0.2121 | 1.01 (0.95 – 1.07) | | 0.7233 |
| Data are presented as percentages (%) or mean ± standard deviation, unless otherwise specified.  Abbreviations:; CABG, coronary artery bypass graft; CAD, coronary artery disease; CHD, coronary heart disease; CVD, cardiovascular disorders; HR, heart rate; NSTE-ACS, non-ST-segment elevation acute coronary syndromes; PCI, percutaneous coronary intervention; SBP, systolic blood pressure | | | | | | |

| **Supplemental Table 7.** Interaction test: calculations for comparing two estimated risk ratios for heart failure on admission (women vs men) by Inverse probability weighting: delay to hospital presentation <120 minutes vs ≥120 minutes in ACS patients | | | |
| --- | --- | --- | --- |
|  | **[Delay to hospital presentation**  **(<120 minutes)]** | **Group 2**  **[Delay to hospital presentation**  **(**≥**120 minutes)]** | ***P-*interaction** |
| All ACS | 1.11(1.04 – 1.19) | 1.14 (1.10 – 1.18) | 0.2457 |
| STEMI | 1.64 (1.51 – 1.77) | 1.24 (1.19 – 1.30) | <0.001 |
| NSTE-ACS | 1.08 (0.96 – 1.21) | 1.01 (0.95 – 1.07) | 0.1564 |
| Abbreviations: ACS, acute coronary syndromes; NSTE-ACS, non-ST-segment elevation acute coronary syndromes; STEMI, ST-segment elevation myocardial infarction | | | |

| **Supplemental Table 8.** Inverse probability weighting in patients with heart failure on admission: clinical factors and outcomes sorted by sex in ACS patients | | | |
| --- | --- | --- | --- |
| **Characteristics** | **Women**  **(N=10,442)** | **Men**  **(N=14,745)** | **Standardized difference** |
| Age (years) | 67.8 ± 11.1 | 68.0 ± 10.9 | -0.02 |
| **Cardiovascular risk factors** |  |  |  |
| Family history of CAD | 42.1 | 41.8 | 0.005 |
| Diabetes | 73.5 | 73.7 | -0.004 |
| Hypertension | 49.5 | 49.7 | -0.005 |
| Hypercholesterolemia | 33.9 | 34.2 | -0.006 |
| Current smokers | 35.0 | 34.7 | 0.007 |
| Former smokers | 1.6 | 1.5 | 0.002 |
| **Clinical history of CHD** |  |  |  |
| Prior stable angina | 34.5 | 34.5 | 0.0006 |
| Prior MI | 25.0 | 24.8 | 0.004 |
| Prior PCI | 6.0 | 5.8 | 0.009 |
| Prior CABG | 4.8 | 4.5 | 0.01 |
| **Clinical history of CVD** |  |  |  |
| Peripheral artery disease | 9.4 | 9.3 | 0.003 |
| Prior stroke | 7.5 | 7.5 | 0.0001 |
| **Clinical presentation on admission** |  |  |  |
| ST-segment shifts in anterior leads (at ECG) | 52.8 | 52.5 | 0.006 |
| SPB at admission, mm Hg | 130.6 ± 30.3 | 130.7 ± 29.3 | -0.003 |
| HR at admission, bpm | 89.8 ± 25.3 | 89.9 ± 25.0 | -0.006 |
| Serum creatinine levels at admission, mg/dL | 1.4 ± 1.3 | 1.4 ± 1.0 | 0.04 |
| **Outcomes** |  |  | ***P* value** |
| 30-day mortality | 29.8 | 25.5 | <0.0001 |
| Risk Ratio (95% CI) | 1.24 (1.17 – 1.31) | | <0.0001 |
| Data are presented as percentages (%) or mean ±standard deviation, unless otherwise specified.  Abbreviations: ACS, acute coronary syndrome; bpm, beats per minute; CABG, coronary artery bypass graft; CAD, coronary artery disease; CHD, coronary heart disease; CVD, cardiovascular disease; HR, heart rate; MI, myocardial infarction; PAD, peripheral artery disease; SBP, systolic blood pressure; | | | |

| **Supplemental Table 9.** Inverse probability weighting in patients without acute heart failure on hospital admission: clinical factors and outcomes sorted by sex in ACS patients. | | | |
| --- | --- | --- | --- |
| **Characteristics** | **Women**  **(n=20,480)** | **Men**  **(n=42,145)** | **Standardized difference** |
| Age (years) | 61.9±11.9 | 61.8±11.5 | 0.009 |
| **Cardiovascular Risk Factors** |  |  |  |
| Family history of CAD | 44.5 | 44.2 | 0.005 |
| Hypercholesterolemia | 22.5 | 22.6 | -0.003 |
| Diabetes | 67.9 | 68.0 | -0.0007 |
| Hypertension | 50.8 | 51.1 | -0.006 |
| Current smoker | 45.3 | 45.3 | -0.0003 |
| Former smoker | 1.9 | 1.9 | -0.003 |
| **Clinical history of CHD** |  |  |  |
| Prior stable angina | 26.8 | 26.2 | 0.01 |
| Prior myocardial infarction | 17.6 | 16.7 | 0.02 |
| Prior PCI | 10.2 | 9.6 | 0.02 |
| Prior CABG | 3.6 | 3.4 | 0.01 |
| **Clinical History of CVD** |  |  |  |
| Peripheral artery disease | 3.9 | 3.6 | 0.01 |
| Prior stroke | 4.1 | 3.9 | 0.009 |
| **Clinical presentation at admission** |  |  |  |
| ST-segment elevation in anterior leads (at ECG) | 39.8 | 39.5 | 0.005 |
| Systolic blood pressure at baseline (mmHg) | 139.4±27.5 | 139.9±26.7 | -0.02 |
| Herat rate at baseline (bpm) | 79.4±19.8 | 79.4±18.6 | 0.0001 |
| Serum creatinine at baseline (mg/dl) | 1.4±1.0 | 1.1±0.7 | 0.11 |
| **Outcome** |  |  | ***P* value** |
| 30-day mortality | 6.7 | 3.3 | <0.0001 |
| Risk Ratio (95% CI) | 2.11 (1.95 – 2.27) | | <0.0001 |
| Data are presented as percentages (%) or mean ± standard deviation, unless otherwise specified.  Abbreviations: ACS, acute coronary syndromes; CABG, coronary artery bypass graft; CAD, coronary artery disease; CHD, coronary heart disease; CVD, cardiovascular disorders; HR, heart rate; PCI, percutaneous coronary intervention; SBP, systolic blood pressure | | | |

| **Supplemental Table 10.** Inverse probability weighting in patients with heart failure on admission who survived till the end of follow up: outcomes sorted by sex in ACS patients | | | |
| --- | --- | --- | --- |
| **Characteristics** | **Women**  **(N=7,316)** | **Men**  **(N=11,022)** | **Standardized difference** |
| Age (years) | 66.9 ± 11.4 | 67.2 ± 11.0 | -0.0306 |
| **Cardiovascular risk factors** | | | |
| Family history of CAD | 43.6 | 43.1 | 0.0089 |
| Diabetes | 75.5 | 75.9 | -0.0101 |
| Hypertension | 52.4 | 53.3 | -0.0182 |
| Hypercholesterolemia | 33.0 | 33.4 | -0.0086 |
| Current smokers | 37.1 | 36.8 | 0.0068 |
| Former smokers | 1.9 | 1.8 | 0.0042 |
| **Clinical history of CHD** | | | |
| Prior angina pectoris | 35.0 | 35.0 | -0.0004 |
| Prior myocardial infarction | 25.1 | 25.3 | -0.0055 |
| Prior PCI | 6.2 | 5.9 | 0.0149 |
| Prior CABG | 5.0 | 4.8 | 0.0113 |
| **Clinical history of CVD** | | | |
| Peripheral artery disease | 8.9 | 8.8 | 0.0028 |
| Prior stroke | 6.4 | 6.5 | -0.0039 |
| **Clinical presentation on admission** | | | |
| ST-segment elevation in anterior leads (at ECG) | 51.7 | 51.2 | 0.0101 |
| SBP at admission (mmHg) | 135.0 ± 28.4 | 135.2 ± 27.7 | -0.0042 |
| HR at admission (bpm) | 87.8 ± 24.0 | 88.0 ± 23.6 | -0.0107 |
| Serum creatinine at admission (mg/dL) | 1.3 ± 1.1 | 1.2 ± 0.8 | 0.0629 |
| **Outcome** |  |  | ***P* value** |
| Ejection Fraction ≤40% | 41.4 | 42.2 | 0.2811 |
| Risk Ratio (95% CI) | 0.97 (0.91 – 1.03) | | 0.2813 |
| Data are presented as percentages (%) or mean ± SD, unless otherwise specified.  Abbreviations: ACS, acute coronary syndromes; CABG, coronary artery bypass graft; CAD, coronary artery disease; CHD, coronary heart disease; CVD, cardiovascular disorders; HR, heart rate; PCI, percutaneous coronary intervention; SBP, systolic blood pressure | | | |

| **Supplemental Table 11.** Inverse probability weighting in patients with heart failure on admission who survived till the end of follow up: outcomes sorted by sex and ACS type | | | | | | |
| --- | --- | --- | --- | --- | --- | --- |
|  | **STEMI** | | | **NSTE-ACS** | | |
| **Characteristics** | **Women**  **(N=4,619)** | **Men**  **(N=7,569)** | **Standardized difference** | **Women**  **(N=2,697)** | **Men**  **(N=3,453)** | **Standardized difference** |
| Age (years) | 65.5 ± 11.9 | 66.1 ± 11.2 | -0.0540 | 69.4 ± 10.2 | 69.4 ± 10.1 | 0.0033 |
| **Cardiovascular risk factors** |  |  |  |  |  |  |
| Family history of CAD | 39.8 | 38.8 | 0.0211 | 51.6 | 51.6 | -0.0001 |
| Diabetes | 71.2 | 72.1 | -0.0193 | 83.8 | 83.5 | 0.0079 |
| Hypertension | 48.5 | 50.1 | -0.0322 | 59.3 | 59.7 | -0.0083 |
| Hypercholesterolemia | 30.5 | 31.1 | -0.0126 | 38.0 | 38.0 | -0.0003 |
| Current smokers | 40.4 | 39.9 | 0.0113 | 30.5 | 30.7 | -0.0053 |
| Former smokers | 1.8 | 1.6 | 0.0141 | 2.1 | 2.2 | -0.0027 |
| **Clinical history of CHD** |  |  |  |  |  |  |
| Prior stable angina | 28.1 | 28.3 | -0.0032 | 48.4 | 48.2 | 0.0033 |
| Prior myocardial infarction | 20.4 | 20.8 | -0.0111 | 34.3 | 34.2 | 0.0006 |
| Prior PCI | 6.1 | 5.3 | 0.0341 | 6.8 | 6.9 | -0.0033 |
| Prior CABG | 3.8 | 3.4 | 0.0195 | 7.4 | 7.4 | 0.0003 |
| **Clinical history of CVD** |  |  |  |  |  |  |
| Peripheral artery disease | 6.8 | 6.6 | 0.0070 | 13.1 | 13.0 | 0.0046 |
| Prior stroke | 5.3 | 5.5 | -0.0105 | 8.7 | 8.5 | 0.0054 |
| **Clinical presentation on admission** |  |  |  |  |  |  |
| ST-segment shifts in anterior leads (at ECG) | 52.4 | 51.3 | 0.0214 | 51.2 | 51.1 | 0.0033 |
| SBP at admission (mmHg) | 133.4 ± 28.3 | 133.7 ± 28.0 | -0.0120 | 138.4 ± 27.4 | 138.2 ± 27.4 | 0.0087 |
| HR at admission (bpm) | 87.6 ± 23.1 | 87.9 ± 23.0 | -0.0153 | 88.1 ± 25.2 | 88.1 ± 25.1 | -0.0029 |
| Serum creatinine at admission (mg/dL) | 1.4 ± 1.4 | 1.2 ± 0.8 | 0.1194 | 1.3 ± 0.9 | 1.3 ± 0.7 | 0.0496 |
| **Outcome** |  |  | ***P* value** |  |  | ***P* value** |
| Ejection Fraction ≤ 40% | 45.3 | 42.4 | 0.0020 | 34.4 | 41.8 | <0.0001 |
| Risk Ratio (95% CI) | 1.12 (1.04 – 1.21) | | 0.0020 | 0.73 (0.66 – 0.81) | | <0.0001 |
| Data are presented as percentages (%) or mean ± SD, unless otherwise specified.  Abbreviations: CABG, coronary artery bypass graft; CAD, coronary artery disease; CHD, coronary heart disease; CVD, cardiovascular disorders; HR, heart rate; PCI, percutaneous coronary intervention; SBP, systolic blood pressure; STEMI, ST-segment elevation myocardial infarction. | | | | | | |

| **Supplemental Table 12.** Interaction test: calculations for comparing two estimated risk ratios for ejection fraction ≤40% (women vs men) in patients with heart failure on admission who survived till the end of follow up by Inverse probability weighting: STEMI vs NSTE-ACS | | | |
| --- | --- | --- | --- |
|  |  | **Group 1**  **[STEMI]**  **(N = 12,188)** | **Group 2**  **[NSTE-ACS]**  **(N = 6,150)** |
| **1** | **RR** | 1.12 | 0.73 |
| **2** | **log RR** | 0.1133 | -0.3147 |
| **3** | **95% CI for RR** | 1.04 – 1.21 | 0.66 – 0.81 |
| **4** | **95%CI for log RR** | 0.039–0.1906 | -0.4155– 0.2107 |
| **5** | **Width of CI** | 0.1514 | 0.2048 |
| **6** | **SE (=width / (2*1.96))** | 0.0386 | 0.0522 |
| **Difference between log risk ratios** | | | |
| **7** | **d (=)** | **0.4280** | |
| **8** | **SE (d)** | **0.0650** | |
| **9** | **CI (d)** | **0.3007 – 0.5554** | |
| **10** | **Test of Interaction** | **6.5883 (p-value <0.0001)** | |
| **Ratio of risk ratios** | | | |
| **11** | **RRR ( =exp(d) )** | 1.5342 | |
| **12** | **CI (RRR)** | 1.3508 – 1.7426 | |

# **REFERENCES:**

1. van Buuren S, Groothuis-Oudshoorn K. mice: Multivariate Imputation by Chained Equations in R. Journal of Statistical Software 2011;45:1 - 67.

2. Austin PC, Stuart EA. Moving towards best practice when using inverse probability of treatment weighting (IPTW) using the propensity score to estimate causal treatment effects in observational studies. Stat Med 2015;34:3661-79.

3. Dongsheng Y, Dalton JE. A unified approach to measuring the effect size between two groups using SAS®: SAS global forum 2012: statistics and data analysis. SAS Global Forum. 2012: 335-2012. Available from: <https://support.sas.com/resources/papers/proceedings12/335-2012.pdf>.

4. Altman DG, Bland JM. Interaction revisited: the difference between two estimates. BMJ 2003;326:219.
